# Global characterization of GH3 family glycoside hydrolase genes in *Fusarium verticillioides* and functional analysis of *FvGH3-6*

**DOI:** 10.3389/fmicb.2025.1543210

**Published:** 2025-03-05

**Authors:** Xiaojie Zhang, Pengliang Duan, Shaoqi Shi, Manli Sun, Ning Liu, Zhiyan Cao, Jingao Dong

**Affiliations:** ^1^State Key Laboratory of North China Crop Improvement and Regulation/College of Plant Protection, Hebei Agricultural University, Baoding, China; ^2^Key Laboratory of Hebei Province for Plant Physiology and Molecular Pathology, Baoding, China

**Keywords:** *Fusarium verticillioides*, glycoside hydrolase 3, expression pattern, functional analysis, pathogenicity

## Abstract

To clarify the roles of glycoside hydrolase 3 (GH3) family genes in the growth, development, and pathogenicity of *Fusarium verticillioides*, GH3 family genes were identified in the genome by bioinformatics software, and their expression levels in the infection process of *F. verticillioides* were analyzed using transcriptome data. The *FvGH3-6* gene was knocked out and complemented via genetic transformation to explore the role of *F. verticillioides*. The results demonstrated that a total of 19 GH3 family genes were identified in the genome of *F. verticillioides*, which were located on 11 chromosomes, encoding amino acids ranging from 559 to 1,034, with relative molecular weights between 61.20 and 113.97 kDa, and containing 1–6 exons. Transcriptome data indicated that during the infection of maize kernels by *F. verticillioides*, the expression of nine genes, including *FvGH3-6*, was upregulated at different stages. Knockout of the *FvGH3-6* gene did not impact the mycelial growth rate of *F. verticillioides* but reduced the sporulation ability. Compared with the wild type, the pathogenicity of *FvGH3-6* knockout mutants towards maize grains and stems was weakened. The above results suggest that the glycoside hydrolase gene family participates in the pathogenicity of *F. verticillioides*, and the *FvGH3-6* gene plays a significant role in the conidia production and pathogenicity of *F. verticillioides*.

## 1 Introduction

Maize (*Zea mays*) is China’s top grain and feed crop. With the advancement of science and technology and the improvement of planting technology, maize production has increased significantly. Nevertheless, diseases continue to impair the yield and quality of maize. *F. verticillioides* has the capability to infiltrate maize via natural apertures and wounds. Seeds harboring *F. verticillioides* are also apt to trigger systemic infections within maize, thereby giving rise to a variety of maize diseases, notably ear rot and stem rot (Blacutt et al., 2018). In recent years, relevant research has shown that *F. verticillioides* spreads widely. The maize ear rot disease caused by this pathogen has now surfaced in multiple countries, such as South Africa, Mexico, Brazil, Canada, Germany, and India (Logrieco et al., 2021; Van et al., 2007). In China, it predominantly appears in key maize-producing areas such as Northeast, North China, and the Huang-Huai-Hai regions (Sun et al., 2017; Logrieco et al., 2021). Diseases induced by *F. verticillioides*, including maize ear rot and stalk rot, demand attention due to their substantial influence on maize yield (Hurni et al., 2015; Mei et al., 2023; Njeru et al., 2023). At the onset of maize ear rot, white mycelium emerges at the ear’s apex. In severe scenarios, it may trigger ear abscission and result in a 30–50% decline in maize yield, or even total crop failure (Yu et al., 2017). Based on Munkvold and Carlton (1997) research, if maize in the field is infected by *F. verticillioides*, 80% of the seeds will ultimately be, 80% of the seeds will ultimately be contaminated. Maize diseases induced by *F. verticillioides* present substantial threats. These diseases not only diminish maize yield but also yield toxic secondary metabolites—fumonisins, which pose a grave peril to the well-being of humans and animals and the security of food and feed (Pienaar et al., 1981). Corn ear rot frequently manifests itself during the late growth phase, and the execution of pesticidal control protocols proves to be considerably arduous. In the wake of the proliferation of mechanized maize harvesting, the mechanical excision of diseased ears becomes challenging, frequently culminating in a commingled harvest with healthy ears, thereby impacting the storage quality and market value of maize (Guo et al., 2023). Meanwhile, *F. verticillioides* is capable of instigating stalk rot in maize, leading to the degeneration of both stalks and leaves. In severe scenarios, lodging is prone to transpire, which erects impediments to mechanized harvesting and incurs significant economic losses in agricultural production (Ding et al., 2023; Marin et al., 2013). Consequently, it is of utmost urgency to undertake a profound exploration of the pathogenic mechanism of *F. verticillioides* and identify the pivotal pathogenic genes so as to establish a theoretical underpinning for the scientific and effective prevention and control of maize diseases.

The glycoside hydrolase (GH) family represents a crucial class of enzymes *in vivo*, playing a significant role in the hydrolysis of diverse glycosidic bonds. Among them, the GH3 family is predominantly composed of *β*-glucosidase (β-D-Glucosidase, EC 3.2.1.21). The function of β-glucosidase involves hydrolyzing the non-reducing β-D-glucosidase bond at the substrate’s terminus, simultaneously releasing *β*-D-glucose and the corresponding ligand (Pan and Luo, 2006). To date, the majority of identified β-glucosidases belong to the GH1 and GH3 families. Notably, numerous fungal-derived β-glucosidases are classified into the GH3 family. In contrast, β-glucosidases from the GH2, GH5, GH16, GH30, GH39, and GH116 families have received relatively little attention in the literature (Ferrara et al., 2014; Qi et al., 2008; Sansenya et al., 2015; Suzuki et al., 2013). *β*-glucosidase is widely distributed in organisms and performs diverse functions across archaea, fungi, and eukaryotes. It participates in a variety of biological processes and metabolic pathways, exerting crucial impacts on organisms. For instance, it is involved in biomass conversion in microorganisms, lignin and cell wall oligosaccharide catabolism, and the decomposition of exogenous glycosidic lipids in animals. Moreover, *β*-glucosidase plays significant roles in plant self-defense mechanisms and plant interactions with microorganisms and insects (Ketudat Cairns and Esen, 2010; Morant et al., 2008). In a previous study, during the infection of apple trees by *Valsa mali*, the β-glucosidase gene *VmGluI* showed a remarkable up-regulation (Li et al., 2017). The knockout of *VmGlu2* was associated with reduced β-glucosidase activity and toxin levels, which in turn led to a decrease in the pathogenicity of *Valsa mali* (Huang et al., 2021). Similarly, knocking out the β-glucosidase gene *Foglu1* in *Fusarium oxysporum f.*sp.*conglutinan* resulted in a reduction of the pathogen’s pathogenicity to cabbage (Wang et al., 2023). Additionally, the knockout of the *pxo04104* gene by *Xanthomonas oryzae pv.oryzae* caused a decline in its pathogenicity to rice and a reduction in the mutant’s osmotic resistance, indicating a positive correlation between the gene and the pathogen’s pathogenicity (Tian et al., 2017). *F. verticillioides*, a phytopathogenic fungus, lacks specialized cellular structures like appressoria and haustoria, which are typically employed by certain pathogens to adhere to and penetrate plant cells. Nevertheless, it is capable of secreting a series of cell wall—degrading enzymes (CWDEs). These enzymes play a crucial role in effectively degrading the components of the plant cell wall, facilitating the fungus’s invasion and nutrient acquisition within the host plant. *β*-glucosidase is among the key CWDEs. In *F. verticillioides*, the functions of β-glucosidase genes belonging to the GH3 family remain unreported to date.

In the present study, we identified the genes within the GH3 family of *F. verticillioides* and analyzed their expression levels. Additionally, we focused on elucidating the functions of the differentially expressed β-glucosidase gene *FvGH3-6*. This was done to uncover its roles in the growth and development of *F. verticillioides*, as well as in the pathogenesis of maize when infected by this fungus. Understanding the functions of *FvGH3-6* holds great significance for exploring the pathogenic mechanisms of diseases caused by *F. verticillioides*. The insights gained from this research can serve as a theoretical foundation for developing more effective strategies for agricultural disease control, aiming to mitigate the damage caused by this pathogen to maize crops.

## 2 Materials and methods

### 2.1 Plants, strains, and culture conditions

*Fusarium verticillioides* 7600 strain was provided by Gu Qin from Nanjing Agricultural University. The plasmid pUCATPH containing hygromycin (HPH) fragment, complementary plasmid pHZ100, and maize B73 were provided by Hebei Key Laboratory of Plant Physiology and Molecular Pathology. The *F. verticillioides* 7600 strain was cultured on potato dextrose agar medium (PDA) in the darkness at 25°C.

### 2.2 Methods

#### 2.2.1 Gene identification and bioinformatics analysis of GH3 gene family in *F. verticillioides*

The genomic sequence of *F. verticillioides* 7600 (ASM14955v1.59) was downloaded from the Ensemble Fungi database,^1^ and the members of the GH3 gene family were screened out from the genomic sequence of *F. verticillioides* 7600 by Hidden Markov Model (HMM). The genes containing the conserved domain (PFAM accession number: PF00933, PF01915) of GH3 protein were screened and identified by the online tool InterPro.^2^ The physical and chemical properties of the protein sequence of the GH3 family of *F. verticillioides* were analyzed using the online website ExPASy.^3^ The gene location and structure were further explored with TBtools (Version 2.154), with all parameters set to default values. The online database WoLF PSORT^4^ was used to predict the subcellular localization of GH3 family members. The phylogenetic tree was constructed using MEGA 7.0 software and the Neighbor-Joining (NJ) method. Bootstrap was repeatedly set to 1, 000, and other parameters were the default values of the system.

#### 2.2.2 Transcriptome analysis of GH3 gene family in *F. verticillioides*

To screen for the key GH3 genes involved in the growth, development, and pathogenicity of *F. verticillioides*, we analyzed the expression levels of GH3 family member genes during different infection stages through transcriptome data and selected genes with significantly upregulated expression levels during the infection process. Meanwhile, the total RNA of *F. verticillioides* mycelia and pathogen—host interaction samples was extracted following the total RNA extraction protocol provided by the miRcute miRNA Extraction and Isolation Kit (Tiangen, Beijing, China). The isolated RNA was stored at −80°C for subsequent studies. cDNA was synthesized by reverse transcription using the PrimeScript™ RT reagent Kit with gDNA Eraser (TaKaRa, Beijing, China). qPCR primers were designed using Primer Premier 5 software. The *Actin* gene of *F. verticillioides* was used as the internal reference gene. Real-time fluorescence quantitative PCR (qPCR) analysis was conducted using the Super EvaGreen qPCR Master Mix Kit. The relative expression levels of genes with significantly upregulated expression in the transcriptome were measured. The samples tested included *F. verticillioides*—infected maize kernels at 4 hpi, 12 hpi, and 72 hpi, and the *in vitro*—cultured mycelia of *F. verticillioides* grown on PDA for 3 days.

#### 2.2.3 The generation of GH3 family gene knockout mutants and complementary strains

Based on the transcriptome gene expression levels described in Section 2.2.2, the key gene *FvGH3-6*, which was significantly up-regulated during infection, was selected to construct knockout mutants and complementary strains. Primers (Supplementary Table S1) were designed by Primer Premier 5.0. The fusion gene fragments required for knockout transformation were obtained by fusion PCR. Mycelia cultured on a PDA plate at 25°C in the dark for 4 days were collected. The DNA of *F. verticillioides* was extracted using the CTAB method and served as a template for PCR amplification. The upstream arm of *FvGH3-6* was amplified using primer 1F/1R to obtain fragment 1. The downstream arm was amplified using primer 2F/2R to obtain fragment 2. The HPH fragment was amplified using primer HPH-F / HPH-R with pUCATPH plasmid as a template, and fragment 3 was obtained. Fragments 1, 2, and 3 were fused based according to the principle of homologous recombination. Using the method described by Müller (1999), the fusion fragments were introduced into the *F. verticillioides* 7600 strain via 40% PEG-mediated protoplast transformation. Using the cDNA of *F. verticillioides* as a template, we amplified the cDNA fragment of the *FvGH3-6* CDS sequence devoid of the stop codon. The amplified fragment was ligated to the pHZ100 plasmid through seamless cloning in accordance with the principle of homologous recombination to generate the complementary vector. These constructs were reintroduced into the respective mutant strains. Complemented strains were selected on G418-containing mediums.

The wild type strains and mutant strains were cultured on a PDA plate at 25°C in the dark for 4 days. DNA was extracted using the CTAB method. The extracted DNA served as a template, and four pairs of primers were used for PCR detection. When no band was detected for the target gene fragment while the other three fragments could be successfully amplified with the correct bands, the knockout mutant was deemed to have been obtained. Two pairs of primers were used to verify the gene complemented transformants. The aminoglycoside phosphotransferase (APH) fragment and *FvGH3-6* target gene were detected, separately. qPCR was used to determine the gene expression levels of the positive transformants. Total RNA was isolated from the mycelium using the Column Fungal Total RNA Extraction and Purification Kit (Sangon Biotech, Shanghai, China). Subsequently, cDNA was synthesized via reverse transcription using the PrimeScript® Reverse Transcription Kit (Takara, Beijing, China) following the manufacturer’s instructions. The *Actin* gene of *F. verticillioides* served as the internal reference gene. The relative expression of each gene was calculated by the 2^−ΔΔCt^ method, and each reaction contained 3 biological replicates.

#### 2.2.4 Analysis of growth and development ability of mutant strains

The samples were collected from the edge of colonies with a diameter of 5 mm of wild-type (WT) strains and mutant strains. Subsequently, they were cultured on PDA plates at 25°C in the dark for 4 days. The colony diameter was then measured and photographed using the cross method. The growth inhibition rate was calculated as follows: (growth diameter of WT—growth diameter of knockout mutant strains) / (growth diameter of WT—5 mm). For this test, three independent biological replicates were established. Additionally, five bacterial cakes with a diameter of 8 mm were, respectively, obtained from the WT and mutant strains and added into 100 mL of CMC liquid medium. The mixtures were incubated at 25°C with shaking at 175 rpm to induce sporulation. After 4 days of culture, the number of conidia was counted, and three independent biological replicates were set up.

#### 2.2.5 Determination of the sensitivity of mutant strains to environmental stress

To explore the growth of gene deletion mutant strains and complemented mutant strains in different stress environments, two stress factors, namely 0.05% sodium dodecyl sulfate (SDS) and 100 μg/mL Congo red (CR), were established. We inoculated three 5 mm diameter fungal cakes of WT strains, knockout mutant strains, and complemented mutant strains on PDA plates containing different stress agents. After culturing in the dark at 25°C and a relative humidity of 60–70% for 5 days, the growth rate of the colony was recorded and the diameter was measured to calculate the growth inhibition rate. Three independent biological replicates were set up.

#### 2.2.6 Determination of pathogenicity of mutant strains

To assess the impact of knocking out the *FvGH3-6* gene on the pathogenicity of *F. verticillioides*, conidia from the WT and knockout mutant strains were used to infect maize kernels and maize stalks. The concentration of the spore suspension was 1 × 10^6^ conidia/mL. The sterile syringe needle gently scratched the endosperm of corn kernels, and each kernel was injected with 15 μL of spore suspension. The corn kernels were cultured in dark and humid conditions at 25 ° C for 3 days. In addition, living maize plants at the 10-leaf stage were injected with 200 μL spore suspension on the third stem node near the ground of live maize. The wound was wrapped with autoclaved absorbent cotton and gauze to prevent contamination by exogenous bacteria. After the inoculated maize stems grew in the field environment for 12 days, a multispectral measurement system was employed to photograph the inoculated and diseased areas. Finally, the relative lesion area was calculated.

#### 2.2.7 Effects of different carbon source conditions on the growth of mutant strains

*β*-glucosidase is an important component of cellulose hydrolase, which can hydrolyze the cellooligosaccharides and cellobiose produced by hydrolysis into glucose (Liu et al., 2022). To explore whether the knockout of the *FvGH3-6* gene affects its ability to hydrolyze cellulose. The mycelial growth of WT and knockout mutant strains under different carbon source conditions was determined, and three carbon sources were selected, namely glucose, cellobiose, and CMC (sodium carboxymethyl cellulose). Three 5 mm diameter fungal cakes of WT and knockout mutant strains were collected and inoculated on the medium containing different carbon sources. After 5 days of culture at 25°C in darkness, with relative humidity of 60–70%, the growth state of the colony was recorded and the diameter was measured. Three independent biological replicates were set up in the experiment.

#### 2.2.8 Statistical analysis

All statistical analyses were performed using SPSS software (Version 26.0) and GraphPad Prism software (Version 9.5). The unpaired *t*-test and one-way analysis of variance were employed to conduct significance tests for differences. Asterisks and different letters indicate statistically significant differences (*p* < 0.05).

## 3 Results

### 3.1 GH3 family members of *F. verticillioides*

**Figure 1:**
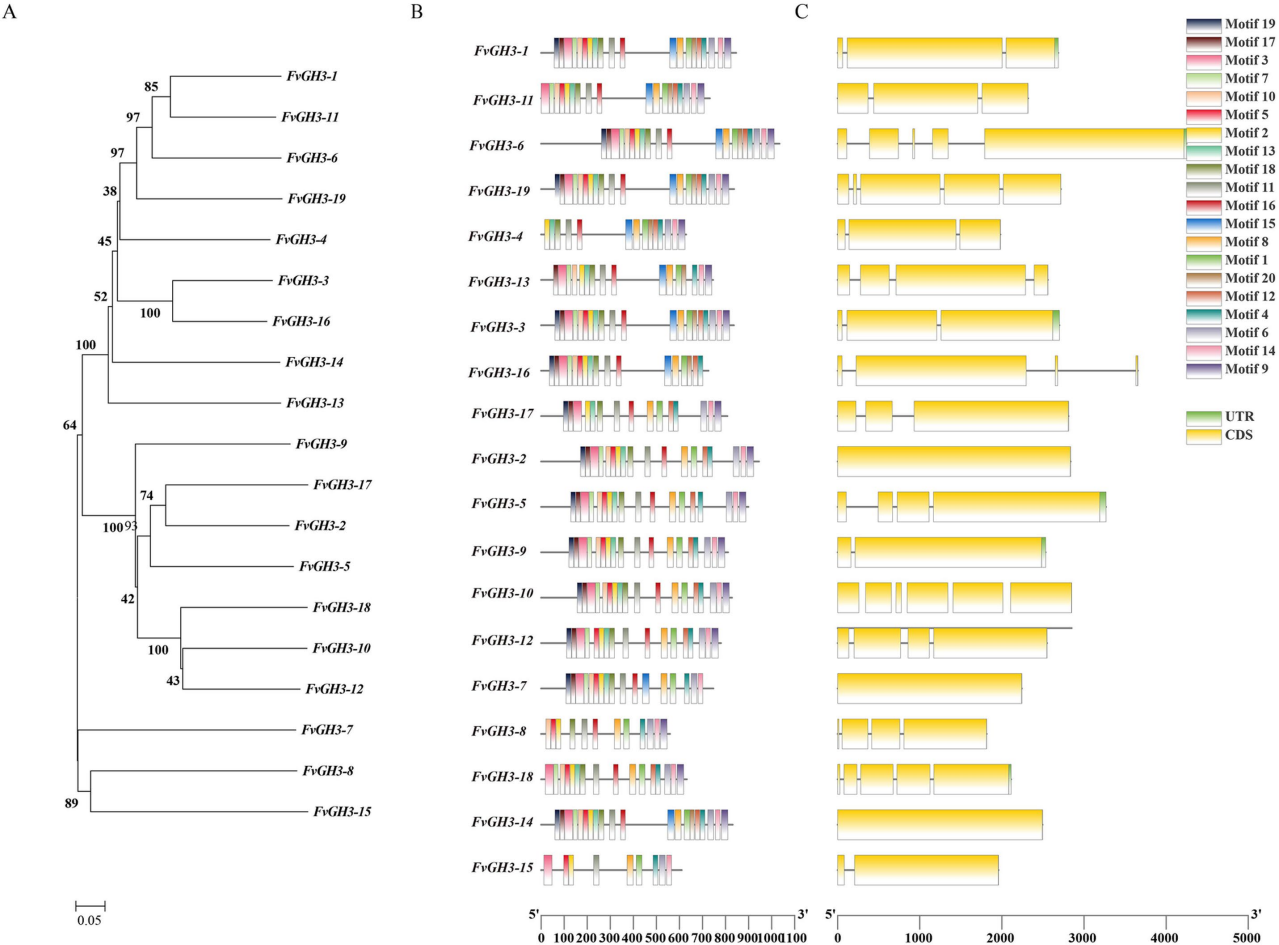
Bioinformatics analysis of GH3 gene family in *F. verticillioides.* **(A)** Phylogenetic tree; **(B)** analysis of amino acid conserved motifs; **(C)** gene structure.

Based on the genome data of *F. verticillioides*, 19 GH3 family genes were successfully screened and identified, and they were numbered *FvGH3-1* ~ *FvGH3-19* according to their location on the chromosome. The 19 genes of the GH3 family were distributed on 11 chromosomes. Which encoding amino acids ranging from 559 to 1,034. The proteins encoded by them, and the relative molecular weight ranged from 61.20 to 113.97 kDa. Among them, *FvGH3-8* encoded the greatest number of amino acids, corresponding to a molecular weight of merely 61.20 kDa. *FvGH3-6* encoded the greatest number of amino acids, corresponding to a molecular weight of 113.97 kDa. Isoelectric points ranged from 4.76 to 6.22, all less than 7. The GH3 protein of *F. verticillioides* were acidic proteins, of which the lowest isoelectric point was at 4.76, while the highest isoelectric point was at 6.22. The instability coefficients of the GH3 proteins of *F. verticillioides* ranged from 26.33 to 43.05, and the instability coefficients of most of the proteins of *F. verticillioides* GH3 family genes were lower than 40, indicating that the proteins encoded by this family of genes were mostly stabilized proteins. Only *FvGH3-4*, *FvGH3-7*, and *FvGH3-14* exhibited instability coefficients higher than 40, which were unstable proteins. The results of subcellular localization showed that 12 FvGH3 members were localized in the cytoplasm, 5 in the extracellular, and 2 in the cytoskeleton (Supplementary Table S2). The phylogenetic tree of the 19 GH3 family proteins of *F. verticillioides* was constructed by MEGA7 software. According to the similarity of amino acid sequences, the phylogenetic tree can be divided into three groups (Figure 1A). To explore the conserved protein motifs of GH3 family members. Tbtools was used to predict the conserved motifs of FvGH3 family members. The results showed that among the 20 conserved motifs, motif 1, motif 3, motif 10, motif 11, and motif 16 were widely present in the 19 GH3 proteins. *FvGH3-8* and *FvGH3-15* contained only 11 motifs, and the other genes had more motifs (Figure 1B). The gene structure of GH3 family members was analyzed and visualized by Tbtools. The number of exons among GH3 family genes varies significantly (Supplementary Table S2), and GH3 family members are composed of 1–6 exons (Figure 1C).

### 3.2 Expression patterns of FvGH3 family genes

**Figure 2:**
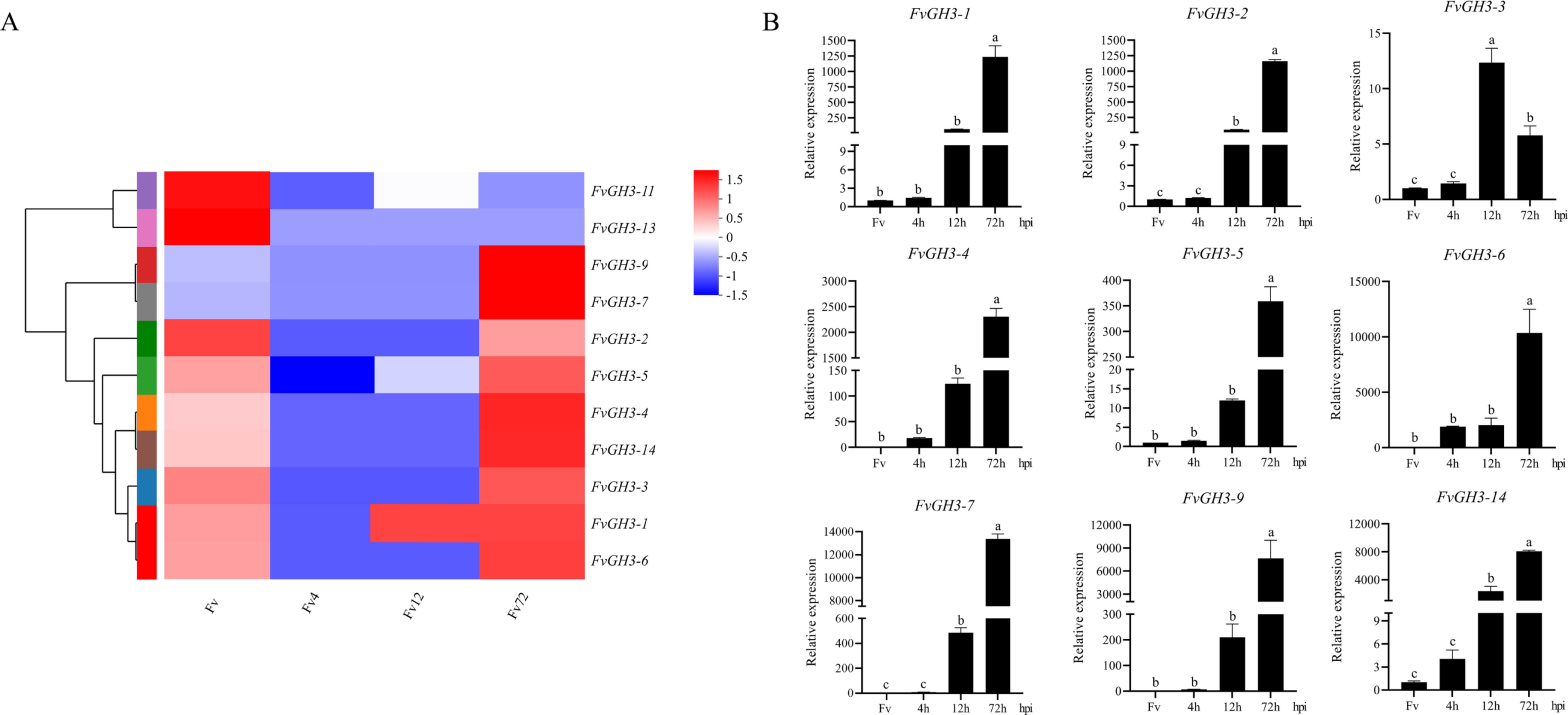
Gene expression of *F. verticillioides* GH3 family members during infection of maize kernels. **(A)** Heatmap of the expression pattern of the GH3 family of *F. verticillioides* during the infection period, **(B)** Validation of the expression levels of the GH3 family of *F. verticillioides* during the infection period by qPCR. Data in the figure are mean ± SE. Different lowercase letters showed significant differences by one-way analysis of variance (*p* < 0.05). hpi, hours post inoculation. The colored bar on the right indicates the gene expression levels. The redder the color, the higher the expression level, and vice versa.

To screen for the key GH3 genes involved in the pathogenesis of *F. verticillioides*, the transcriptome data of pathogen samples at the infection stages of 4 hpi, 12 hpi, and 72 hpi, and the *in vitro* cultured mycelia of *F. verticillioides* grown on PDA for 3 days was used. Subsequently, a heatmap of the expression patterns of FvGH3 gene family members during the infection stages was drawn. Our study revealed that, compared with the *in vitro*—cultured mycelium of *F. verticillioides* that had been cultured on PDA for 3 days and the samples at 4 hpi of the pathogen, the relative expression levels of *FvGH3-1*, *FvGH3-2*, *FvGH3-3*, *FvGH3-4*, *FvGH3-5*, *FvGH3-6*, *FvGH3-7*, *FvGH3-9*, and *FvGH3-14* in the GH3 gene family of *F. verticillioides* at 72 hpi increased significantly. Two genes, *FvGH3-11* and *FvGH3-13*, exhibited inverse expression patterns. Moreover, the other eight genes remained unexpressed during the infection process (Figure 2A). Therefore, we conducted qPCR analysis for the nine genes whose expression levels were up-regulated during the infection period. qPCR was performed on maize kernel samples at 4 hpi, 12 hpi, and 72 hpi after *F. verticillioides* infestation and on *F. verticillioides* mycelium cultured for 3 days. Results showed these nine genes were up-regulated during the infestation period. Specifically, the relative expression of *FvGH3-3* was highest at 12 hpi. The relative expression of the other eight genes gradually increased as the infestation time extended, and the highest relative expression was observed at 72 hpi (Figure 2B). *FvGH3-6* had the highest relative expression during infestation, therefore, the *FvCH3-6* gene was finally selected to construct knockout mutant strains and complementary mutant strains to analyze the function of the GH3 family.

### 3.3 Acquisition of *FvGH3-6* knockout mutants and complementary strains

**Figure 3:**
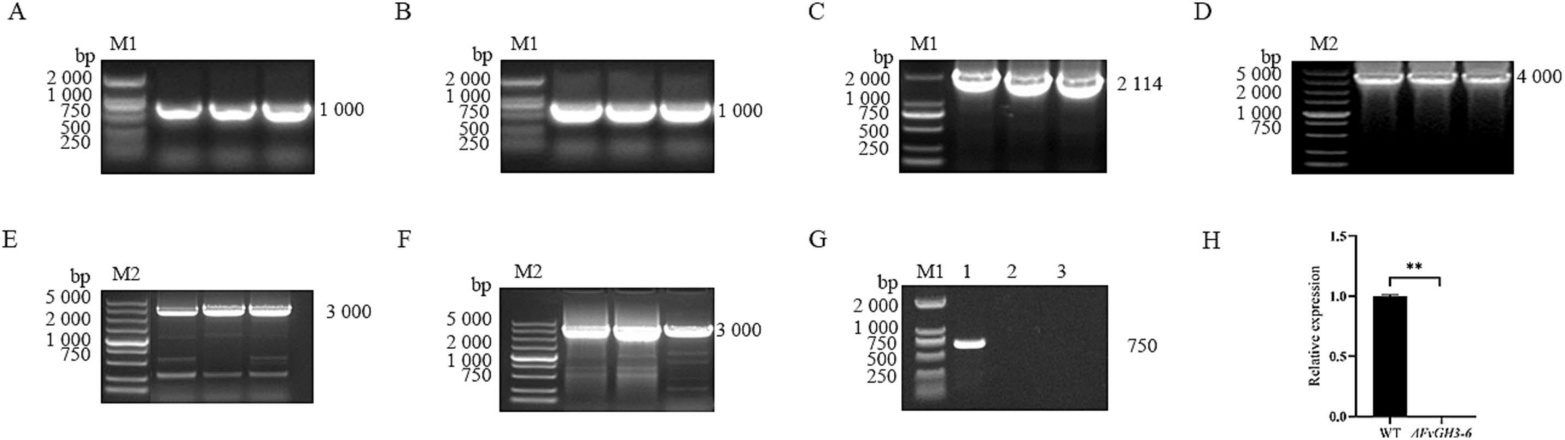
Identification of *F. verticillioides FvGH3-6* gene knockout mutant strain PCR and qPCR. **(A,B)** The upstream and downstream homologous arms of the *FvGH3-6* gene were amplified; **(C)** Amplification of hygromycin fragment containing promoter in pUCATPH plasmid; **(D)** Fusion fragment; **(E–G)** Validation of the target gene fragment by PCR; **(H)** The expression level of *FvGH3-6* gene in knockout mutants was detected by qPCR. M1: 2,000 bp marker; M2: 5,000 bp marker; 1: wild type; 2: negative control; 3: *∆FvGH3-6*. Data in the figure are mean ± SE in **H**. ^**^ indicates significant difference by *t*-test (*p* < 0.01).

**Figure 4:**
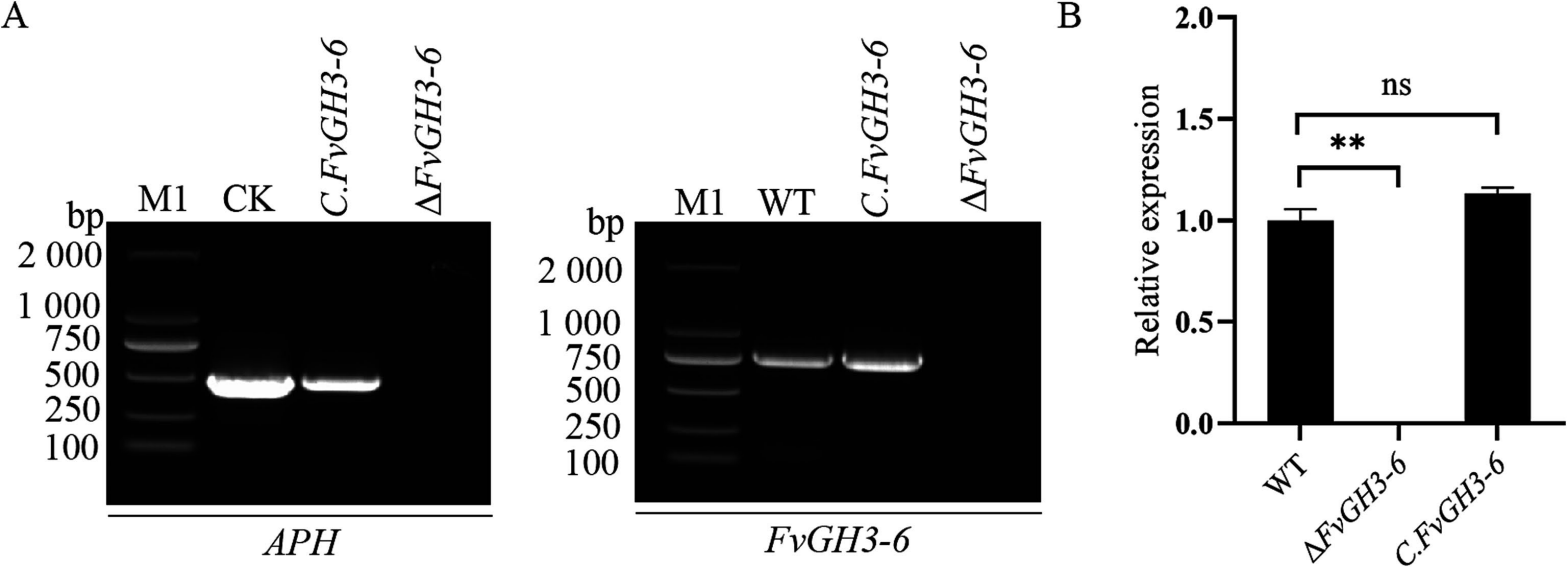
Identification of *F. verticillioides FvGH3-6* gene complemented mutant strain PCR **(A)** and qPCR **(B)**. **(A)** The *FvGH3-6* gene and *APH* gene were verified by PCR; **(B)** The expression level of the *FvGH3-6* gene in the complemented mutant was detected by qPCR. M1: 2,000 bp marker; WT: wild type; *∆FvGH3-6*: gene knockout mutant; CK: pHZ100 plasmid; *C. FvGH3-6*: complemented mutant; APH: aminoglycoside phosphotransferase. Data in the figure are mean ± SE in **B**. ^**^ indicates significant difference by *t*-test (*p* < 0.01), ns indicates no significant difference (*p* > 0.05).

By PCR, we verified the target gene fragment of the knockout transformants exhibiting stable hygromycin resistance, and this verification demonstrated the absence of the target gene in the mutants (Figures 3A–G). Subsequently, we employed qPCR for the detection of the *FvGH3-6* gene expression level in the knockout mutants and found that the target gene expression level was significantly reduced (Figure 3H). These results suggest the successful generation of the *FvGH3-6* gene knockout mutants. We then introduced the complementation vector into the protoplasts of the knockout strain, resulting in the generation of the complementation mutant strains. PCR-based verification of the generated complementation transformants validated the presence of the *FvGH3-6* gene and the *APH* gene in the complementation strains (Figure 4A). Subsequently, qPCR was employed for assessing the *FvGH3-6* gene expression level in the complementation mutant strains (Figure 4B), and the target gene had restored the WT gene expression level. These results suggest the successful generation of the *FvGH3-6* gene complementation mutants.

### 3.4 *FvGH3-6* affected the pathogenicity of *F. verticillioides* to maize grain and stem

**Figure 5:**
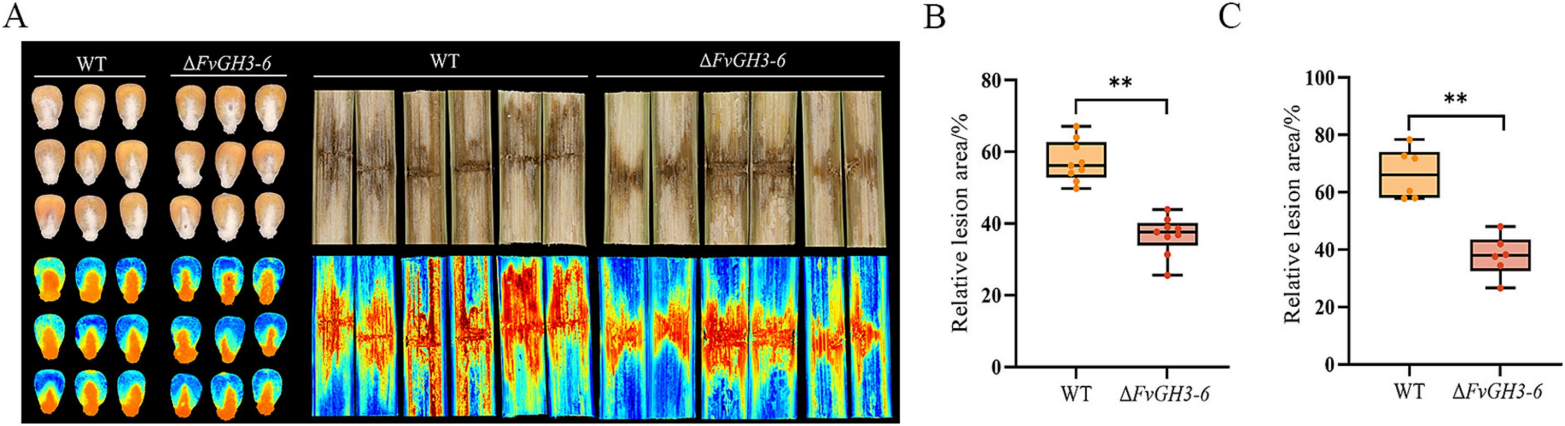
The symptoms of maize kernels and stems **(A)** and the relative lesion area in maize kernels **(B)** and stems **(C)** of *FvGH3-6* gene knockout mutants of *F. verticillioides*. Data in the figure are mean ± SE in **(A, B)**. ^**^ indicates significant difference by *t*-test (*p* < 0.01).

The WT and the mutant Δ*FvGH3-6* were inoculated onto maize seeds and stems to detect the effect of gene knockout on the pathogenicity of maize. The results showed that compared with the WT, the relative lesion area of maize kernels and stems after inoculation with the Δ*FvGH3-6* strain was smaller (Figure 5A). Compared with the WT, the lesion area of maize kernels inoculated with the Δ*FvGH3-6* strain decreased significantly by 20.66% (Figure 5B), and the lesion area of maize stems inoculated with the Δ*FvGH3-6* strain decreased significantly by 28.72% (Figure 5C). These results indicate that the knockout of the *FvGH3-6* gene led to a significant decrease in the pathogenicity of the pathogen. Additionally, they suggest that *FvGH3-6* plays a crucial role in the pathogenicity of *F. verticillioides*.

### 3.5 *FvGH3-6* affected the growth, development and stress resistance of *F. verticillioides*

**Figure 6:**
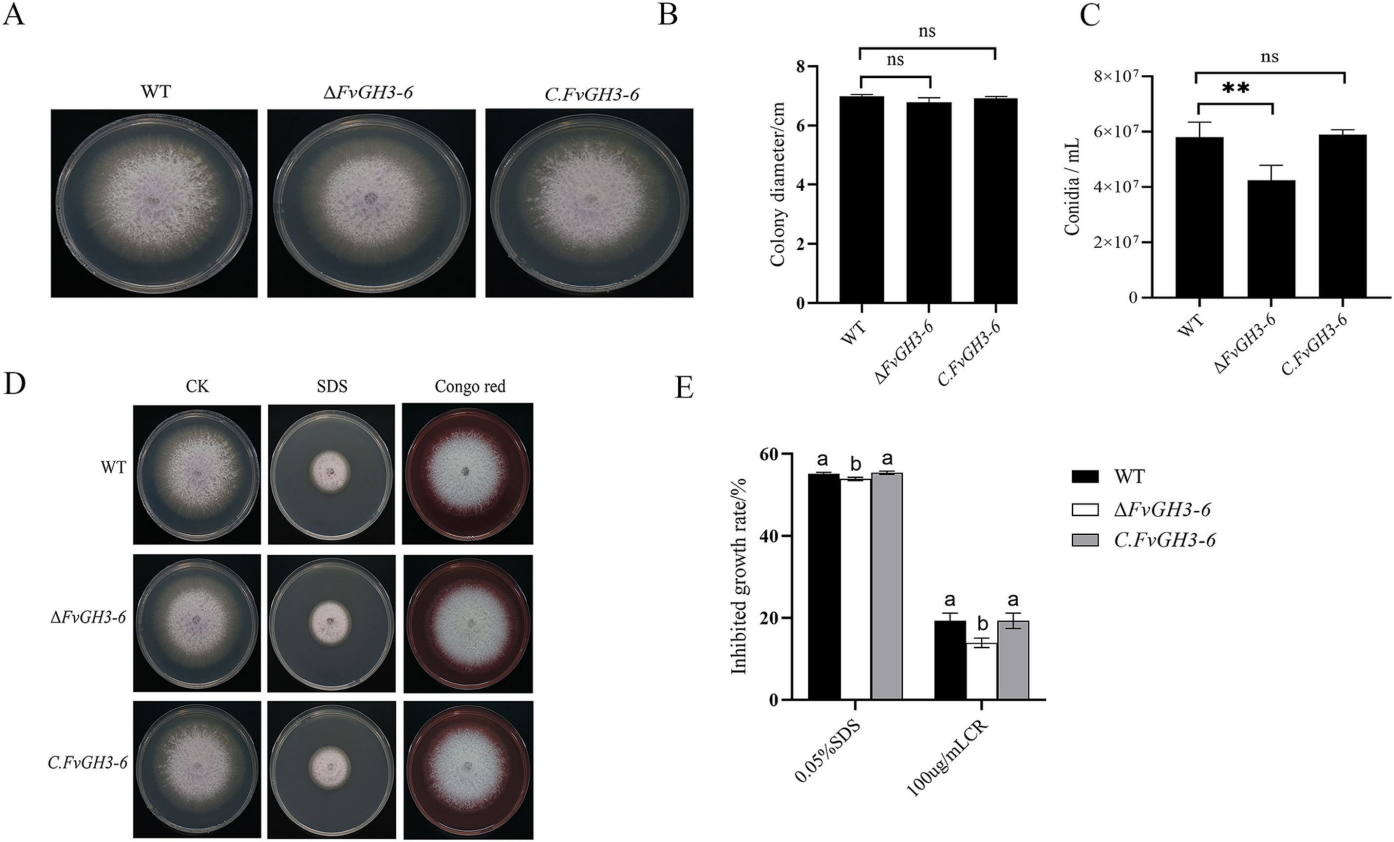
The growth phenotypes of the *FvGH3-6* gene knockout mutant strain and complemented strain in *F. verticillioides* are presented here. **(A,B)** The growth phenotypes and diameters of the mutants after 4 days of growth on PDA. **(C)** The conidiospore production of the mutants. **(D,E)** The growth status of the mutants on PDA media supplemented with different stress agents. Data in the figure are presented as the mean ± SE. “^**^” indicates a significant difference (*p* < 0.01) as determined by the *t*-test, while “ns” indicates no significant difference (*p* > 0.05). Columns with different letters represent significant differences at *p* < 0.05.

An analysis was conducted on the phenotype and growth rate of the mutants. It was observed that there was no significant difference in the colony morphology between the mutants and the WT (Figure 6A), and the growth rate was not affected as well (Figure 6B). However, the conidia production of the knockout mutant strain Δ*FvGH3*-6 decreased, exhibiting a 26.9% reduction in comparison with that of the WT. In contrast, no significant difference was detected in the conidia production of the complemented strain *C. FvGH3-6* relative to that of the WT (Figure 6C). The result indicates that *FvGH3-6* affects the sporulation ability.

We assessed the effects of cell wall-disrupting agents on the growth of the Δ*FvGH3*-6 mutant strain (Figures 6D,E). The growth inhibition rates of the knockout mutant Δ*FvGH3-6* under SDS and CR treatments were 53.92 and 13.92%, respectively, both of which were significantly lower than those of the WT. The deletion of the *FvGH3-6* gene resulted in an enhanced resistance to the fungal cell wall inhibitors CR and SDS. This indicates that *FvGH3-6* affects the cell wall synthesis of *F. verticillioides*.

### 3.6 *FvGH3-6* exhibits no significant effect on the utilization of carbon sources of *F. verticillioides*

**Figure 7:**
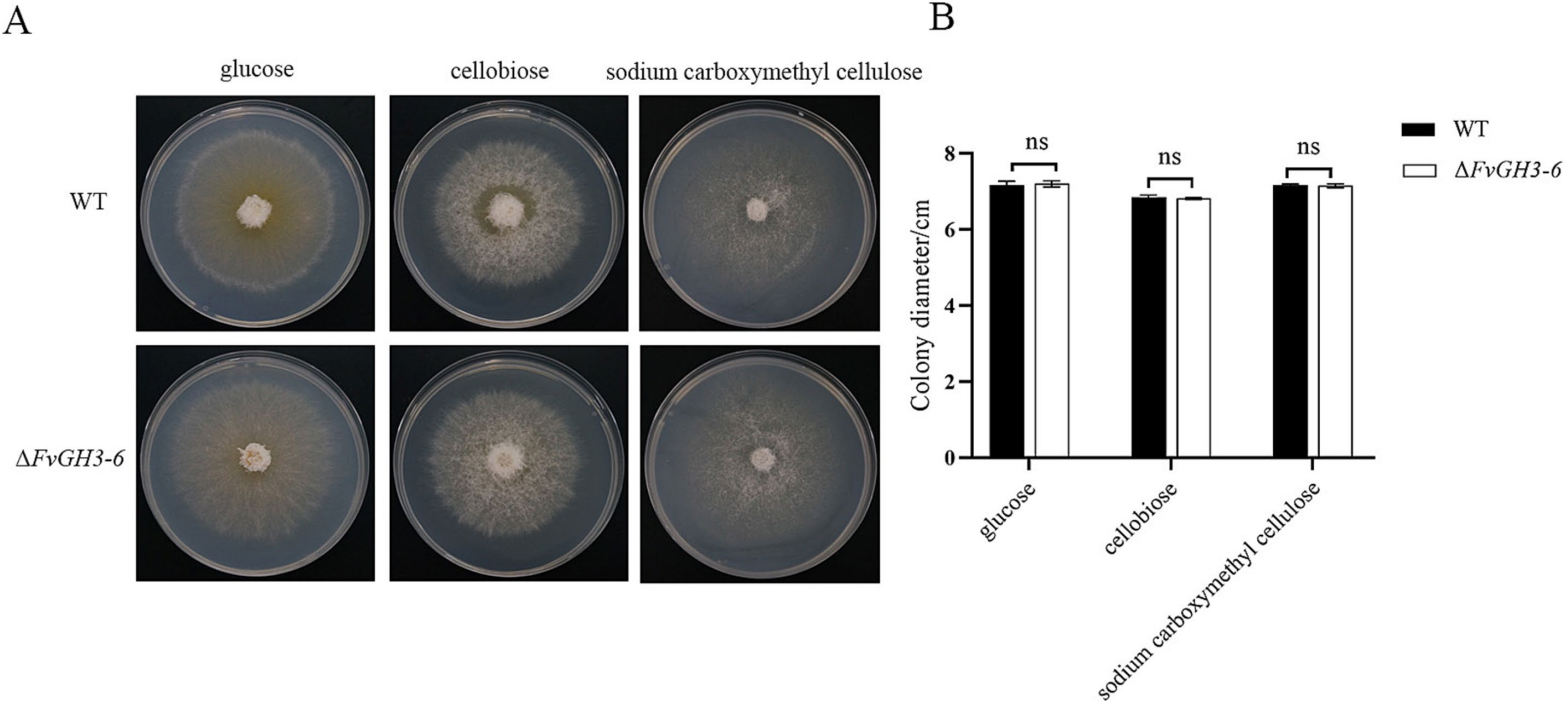
Growth of *F. verticillioides* wild-type and *FvGH3-6* gene knockout mutant strain on media with different carbon sources added. **(A)** Colony phenotype diagram, **(B)** Colony growth diameter. Data in the figure are mean ± SE. ^**^ indicates a significant difference by *t*-test (*p* < 0.01), ns indicates no significant difference (*p* > 0.05).

The WT and mutant strain Δ*FvGH3-6* were cultured under different carbon source conditions, and the effect of gene knockout on carbon source utilization ability was detected through the measurement of mycelial growth (Figure 7A). The results showed no significant difference in mycelial growth between WT and mutant strain Δ*FvGH3-6* on the medium containing glucose, cellobiose, and sodium carboxymethyl cellulose as individual carbon sources. These results suggested that the deletion of the *FvGH3-6* gene did not affect the utilization capacity of the strain to cellobiose and sodium carboxymethyl cellulose (Figure 7B).

## 4 Discussion

GH is a large class of hydrolases that act on different carbohydrate substrates and play a variety of roles. As a common *β*-glucosidase, GH3 has a large number of family members in organisms. Previous studies reported that 13 GH3 family genes were screened from the whole genome of *Dendrobium officinale*, and the expression patterns of these family genes were significantly different in different tissues and treatments (Li et al., 2024). Fungi also possess a large number of GH3 family genes. *Aspergillus niger* harbors 11 genes belonging to the GH3 family, which possess potential β-glucosidase functions (Pel et al., 2007). Researchers screened and identified 16 GH3 gene family members from the genome of *Aspergillus aculeatus*, and the expression levels of these members under cellulose induction were in line with the changes in extracellular β-glucosidase activity (Li and Wang, 2023). The genome of *Neurospora crassa* contains seven β-glucosidase genes, *gh1-1*, *gh3-1* ~ *gh3-6*, belonging to the GH1 and GH3 families. These genes encode proteins that play important roles in the degradation of cellulose (Galagan et al., 2003; Wu et al., 2013; Zhang et al., 2024). According to the amino acid sequence homology, our research identified that there were a total of 19 GH3 family genes in the genome of *F. verticillioides*, distributed on 11 chromosomes, and all these genes contained the typical domain of the CH3 family. However, the functions of these genes remain unclear. As the corn diseases caused by *F. verticillioides* have become increasingly severe, we focused on the role of GH3 family genes in pathogen infection. In this study, transcriptional screening was performed at the transcriptional level. We observed that during the infection of maize kernels by *F. verticillioides*, as the infection time elapsed, the expression levels of nine GH3 gene family members increased significantly, indicating that multiple genes of the GH3 family were involved in the pathogenic process of *F. verticillioides* and might play an important role therein.

The integrity of the fungal cell wall plays a pivotal role in the life process of fungi. On the one hand, it is crucial for fungi to successfully infect host cells, as it can maintain the inherent shape of fungal cells and precisely regulate the interactions between fungal cells and the complex external environment (Cabib et al., 2001). On the other hand, the continuous process of cell wall remodeling is also a necessary condition for ensuring the normal growth and development of fungi (Bowman and Free, 2006). Numerous research examples have highlighted the profound impacts of cell wall-related genes on fungal characteristics. In *Neostagonosporella sichuanesis*, through in-depth investigation, our research ascertained that the Δ*Nsxyn1* and Δ*Nsxyn2* strains exhibited extremely significant sensitivity when faced with cell wall stressors (Liu et al., 2024), which clearly revealed the close association between the deletion of relevant genes and the cell wall stress response. Similarly, in the research field of *Aspergillus fumigatus*, the *edeA* deletion mutant was confirmed to have a substantially increased sensitivity to cell wall-disrupting agents, fully indicating that *EdeA* plays an indispensable and crucial role in maintaining the integrity of the cell wall of *A. fumigatus* (Dai et al., 2024). Further focusing on the research object *F. verticillioides*, the connection between its GH family genes and cell wall characteristics has gradually emerged. For example, after knocking out the *FvGH16-2* gene of *F. verticillioides*, the sensitivity of the mutant to cell wall inhibitors CFW and CR increased significantly, consequently resulting in the impairment of the integrity and functionality of the fungal cell wall (Ren et al., 2024). In the paper, after the knockout of the *FvGH3-6* gene of *F. verticillioides*, the resistance of the mutant to cell wall inhibitors CR and SDS was enhanced, indicating that *FvGH3-6* exerted a significant influence on the integrity and function of the cell wall of *F. verticillioides.*

The production of conidia is a crucial stage in the life cycle of pathogens. Therefore, it is widely believed that inhibiting conidia formation is presumed to mitigate or proficiently regulate the occurrence of diseases. However, the relationship between conidia and the pathogenicity of pathogens remains unclear. In a previous study, the deletion of transcription factors *FvMbp1* and *FvSwi6* severely affected the growth rate of the pathogen and the number of conidia produced. Meanwhile, the virulence on maize stalks and ears also decreased significantly (Huang et al., 2024). In a previous study, the deletion of *VmE02*, encoding the pathogen-associated molecular pattern (PAMP), demonstrated attenuated conidiation but not the attenuation of virulence (Nie et al., 2019). The deletion mutant of *VmGlu2* exhibited a normal growth rate but significantly reduced pycnidia formation and pathogenicity (Huang et al., 2021). In this study, the Δ*FvGH3-6* mutant showed a normal growth rate, decreased conidia production, and reduced pathogenicity. These results suggest that the production of conidia in pathogens is not necessarily related to the growth rate and virulence.

For fungi to infect plants, they must traverse the plant cell wall, which is an important barrier for plants to resist pathogen attack. Many scholars regard the ability of fungi to destroy plant tissue structure during infection as an important criterion for judging whether they are pathogenic (Marie et al., 2000). GH has been reported to be closely related to the pathogenicity of plant pathogenic fungi. It can release nutrients and enhance the permeability of mycelium by hydrolyzing carbohydrates in the plant cell wall, such as cellulose, hemicellulose, and lignin, to promote the infection of host plants by pathogenic bacteria (Han and Xu, 2017). In *Penicillium expansum*, the deletion of *β*-glucosidase 1b (*eglB*), a member of the glycoside hydrolase family, resulted in a reduction in the growth of fungal hyphae, a decrease in the ability of *P. expansum* to produce spores and patulin, and a decrease in pathogenicity to pears (Xu et al., 2024). Upon knockout of the glycoside hydrolase family *FvGH16-2* gene of *F. verticillioides*, the pathogenicity of *F. verticillioides* to maize grains and stems was attenuated (Ren et al., 2024). The deletion of *VmGluI* and *VmGlu2* resulted in a reduction in the infectivity of *V. mali* (Huang et al., 2021; Li et al., 2017). The results demonstrated that the knockout of the *FvGH3-6* gene resulted in a significant decrease in the pathogenicity of *F. verticillioides* to maize grains and stems, indicating that this gene affected the pathogenicity of *F. verticillioides* and played significant roles in plant-pathogen interaction. Numerous reports have indicated that the GH3 family is involved in the degradation of cellobiose and cellulose (Liu et al., 2022; Singhania et al., 2013). Similar to the case when glucose was used as a carbon source, the growth rate of the *F. verticillioides* mutant with the *FvGH3-6* gene knocked out was comparable to that of the WT in media containing cellobiose or sodium carboxymethyl cellulose, indicating that the deletion of the *FvGH3-6* gene did not affect the ability of the strain to degrade cellulose. Therefore, the effect of *FvGH3-6* on the pathogen’s pathogenicity is regulated by a more complex mechanism, and the function of *FvGH3-6* in the interaction between maize and *F. verticillioides* remains to be further studied.

Currently, biocontrol agents and chemical fungicides have been used to mitigate the occurrence of maize ear rot. However, the problem of drug resistance is becoming increasingly severe. Therefore, it is necessary to conduct in-depth research on the pathogenic mechanism of *F. verticillioides* and develop new and highly effective fungicides thereon to prevent the epidemic of this disease. In this study, the knockout of the *FvGH3-6* gene affected the pathogenicity of *F. verticillioides*, and this gene may potentially serve as a molecular target for fungicides to prevent the occurrence of the disease.

## 5 Conclusion

In summary, this study preliminarily analyzed the basic situation of the GH3 family of *F. verticillioides*, and analyzed the biological functions of the significantly up-regulated *FvGH3-6* gene in the growth, development, and pathogenicity of *F. verticillioides*, which provided theoretical support for further in-depth study of the role of *F. verticillioides* in growth, development and pathogenicity, and provided a theoretical basis for scientific and effective prevention and control of maize diseases.

## Data Availability

The original contributions presented in the study are included in the article/Supplementary material, further inquiries can be directed to the corresponding authors.

## Footnotes

^1^ https://fungi.ensembl.org/index.html

^2^ https://www.ebi.ac.uk/interpro/

^3^ https://web.expasy.org/protparam/

^4^ https://wolfpsort.hgc.jp
