# Supplementary material for: Global characterization of GH3 family glycoside hydrolase genes in Fusarium verticillioides and functional analysis of FvGH3-6
Source: Front Microbiol. 2025 Mar 5;16:1543210. doi: 10.3389/fmicb.2025.1543210 (PMC11919885; doi:10.3389/fmicb.2025.1543210)
Supplement: Supplementary file 1 [file Table_1.docx]

Supplementary Material

Supplementary Table S1 Primers information used in this study

| Name | Sequence (5'-3') | Purpose |
| --- | --- | --- |
| FVEG_09248-1F | GCCGTGTCAGTCGTCAGTAT | Knockout and complement primers |
| FVEG_09248-1R | GCTCCTTCAATATCATCTTCTGATGATCAATACCCGAGATTTCCAC |  |
| FVEG_09248-HPH-F | GTGGAAATCTCGGGTATTGATCATCAGAAGATGATATTGAAGGAGC |  |
| FVEG_09248-HPH-R | CAAGATGCTACTGACAACATGAAAGAAGGATTACCTCTAAACAA |  |
| FVEG_09248-2F | TTGTTTAGAGGTAATCCTTCTTTCATGTTGTCAGTAGCATCTTG |  |
| FVEG_09248-2R | CGACTTTGACGGACGATT |  |
| H-F | CAGAAGATGATATTGAAGGA |  |
| H-R | AAAGAAGGATTACCTCTAAAC |  |
| FVEG_09248-F | TGCCTACACCCATAAACTGA |  |
| FVEG_09248-R | CACCATACCACGCCTGAAT |  |
| HF-F | CGCGGTGGCGGCCGCTCTAGAATGGCTGACATTAACGTCG |  |
| HF-R | CTTGCTCACCCTATCGAATTCAATACCAGACCACCAGTATG |  |
| PHZ100-APH-F  PHZ100-APH-R | GCGGCGATACCGTAAAGCA  ACTGAAGCGGGAAGGGACT |  |
| Actin-F | TGCTCCTGAGGCTCTCTTCCA | qPCR |
| Actin-R | AAGCAAGAATAGAACCACCGA |  |
| Fv-09248qPCR-F | CTTTGGTCACGGCCTCTCAT |  |
| Fv-09248qPCR-R | CCTTTGCGAAACCCTTGAGC |  |
| Fv-00180qPCR-F | ATGAGTATGCCTGGCGATGG |  |
| Fv-00180qPCR-R | CTAACTCGGCGTGATCCTCC |  |
| Fv-05521qPCR-F | TCGCCGCCTCATTATCCTTC |  |
| Fv-05521qPCR-R | AAAGCGCTGTTGTAGTCGGA |  |
| Fv-13301qPCR-F | TACGAAACGCTGCGACTTCT |  |
| Fv-13301qPCR-R | CTGGGTGCGCAAAATGTTCA |  |
| Fv-06728qPCR-F | GCTACCACCAACGTCACTGA |  |
| Fv-06728qPCR-R | GTGAGTGACGACGACAGTGT |  |
| Fv-08365qPCR-F | GGAAGCCTGGCCTACGTATC |  |
| Fv-08365qPCR-R | TCGCTCAATCTTGGCCTTGT |  |
| Fv-00180qPCR-F | TCACTTCTTCGACAGCACCC |  |
| Fv-00180qPCR-F | CGCCATTCTTTTGCACACCA |  |
| Fv-03574qPCR-F | GACGCTGGTGGAAAAGGTCT |  |
| Fv-03574qPCR-R | TCCGCAAGGGAATAGTGCAG |  |
| Fv-00412qPCR-F | ACAAGCTCGCTATGCACACT |  |
| Fv-00412qPCR-R | GCTCGTGACCAGTGAAGACA |  |

Supplementary Table S2 Basic information of GH3 family members of *Fusarium verticillioides*

| Gene name | Gene ID | Chromosome | Exons | Amino acid length | Molecular weight | Theoretical pI | Instability index | Subcellular localization |
| --- | --- | --- | --- | --- | --- | --- | --- | --- |
| *FvGH3-1* | FVEG_00118 | chr01 | 3 | 846 | 92.25 | 5.46 | 36.14 | cytoskeleton |
| *FvGH3-2* | FVEG_00180 | chr01 | 1 | 945 | 102.80 | 5.67 | 31.75 | cytoplasm |
| *FvGH3-3* | FVEG_00412 | chr01 | 3 | 836 | 90.74 | 5.35 | 31.05 | cytoplasm |
| *FvGH3-4* | FVEG_03574 | chr02 | 3 | 629 | 70.00 | 5.13 | 42.19 | cytoplasm |
| *FvGH3-5* | FVEG_05521 | chr03 | 4 | 899 | 96.93 | 5.27 | 32.09 | extracellular |
| *FvGH3-6* | FVEG_09248 | chr05 | 4 | 1034 | 113.97 | 5.52 | 34.86 | cytoplasm |
| *FvGH3-7* | FVEG_13301 | chr06 | 5 | 747 | 82.35 | 5.59 | 43.05 | cytoplasm |
| *FvGH3-8* | FVEG_02058 | chr06 | 4 | 559 | 61.20 | 5.48 | 26.33 | cytoplasm |
| *FvGH3-9* | FVEG_06728 | chr07 | 2 | 811 | 88.40 | 4.89 | 28.03 | extracellular |
| *FvGH3-10* | FVEG_11749 | chr07 | 6 | 828 | 89.58 | 5.85 | 33.40 | extracellular |
| *FvGH3-11* | FVEG_07762 | chr08 | 3 | 732 | 80.62 | 5.78 | 37.02 | cytoplasm |
| *FvGH3-12* | FVEG_13391 | chr08 | 4 | 780 | 83.67 | 5.16 | 35.32 | extracellular |
| *FvGH3-13* | FVEG_10024 | chr09 | 4 | 746 | 81.60 | 5.23 | 37.24 | cytoplasm |
| *FvGH3-14* | FVEG_08365 | chr10 | 1 | 831 | 91.90 | 5.32 | 40.99 | cytoplasm |
| *FvGH3-15* | FVEG_08650 | chr10 | 2 | 610 | 65.93 | 6.22 | 33.12 | cytoplasm |
| *FvGH3-16* | FVEG_08842 | chr10 | 4 | 726 | 79.09 | 5.33 | 35.27 | cytoplasm |
| *FvGH3-17* | FVEG_08890 | chr10 | 3 | 808 | 87.52 | 5.27 | 29.11 | extracellular |
| *FvGH3-18* | FVEG_13055 | chr11 | 5 | 632 | 69.13 | 4.76 | 32.45 | cytoplasm |
| *FvGH3-19* | FVEG_10788 | chr11 | 5 | 837 | 92.11 | 5.30 | 32.73 | cytoskeleton |
